# Mid-Term Outcomes of Titanium Plate Reconstruction for Lung Cancer Requiring Chest Wall Resection

**DOI:** 10.1093/icvts/ivag131

**Published:** 2026-05-04

**Authors:** Ryu Kanzaki, Shuhei Kobayashi, Daiki Hayashi, Kensuke Kojima, Toshiteru Tokunaga, Hyungeun Yoon

**Affiliations:** Department of General Thoracic Surgery, NHO Kinki Chuo Chest Medical Center, Sakai, Japan; Department of General Thoracic Surgery, NHO Kinki Chuo Chest Medical Center, Sakai, Japan; Department of General Thoracic Surgery, NHO Kinki Chuo Chest Medical Center, Sakai, Japan; Department of General Thoracic Surgery, NHO Kinki Chuo Chest Medical Center, Sakai, Japan; Department of General Thoracic Surgery, NHO Kinki Chuo Chest Medical Center, Sakai, Japan; Department of General Thoracic Surgery, NHO Kinki Chuo Chest Medical Center, Sakai, Japan

**Keywords:** NSCLC, surgery, chest wall reconstruction, titanium plate

## Abstract

**Objectives:**

Chest wall resection for non-small cell lung cancer (NSCLC) with chest wall invasion is rare and technically demanding, requiring complete resection and durable reconstruction. Although titanium-based devices are increasingly used, NSCLC-specific outcome data remain limited. We evaluated short- and mid-term outcomes of titanium plate-based chest wall reconstruction in NSCLC.

**Methods:**

We retrospectively reviewed 16 consecutive patients who underwent en bloc pulmonary and chest wall resection with titanium plate reconstruction for NSCLC between 2013 and 2019. Indications included resection of ≥3 ribs or anticipated instability. Reconstruction used perforated titanium plates tailored to defect geometry. Postoperative complications, late reconstruction-related events, and oncologic outcomes were analysed.

**Results:**

Preoperative therapy was administered in 8 patients (50%), including chemoradiotherapy in 7. R0 resection was achieved in 94%. Defects were predominantly posterior, with reconstruction adjacent to the scapula in 12 patients (75%). Thirty-day postoperative complications occurred in 9 patients (56%), consisting predominantly of pulmonary events. Postoperative chemotherapy was given to 6 patients (38%). During a median follow-up of 24 months (mean 54 months), 2 late complications occurred: chest wall haematoma and empyema. Only 1 chest wall local recurrence was observed. No structural failure or scapular impingement occurred. Five-year recurrence-free and overall survival rates were 35% and 63%, respectively.

**Conclusions:**

Titanium plate-based chest wall reconstruction enables extensive en bloc resection for NSCLC with chest wall invasion while maintaining mid-term structural stability. With appropriate patient selection and follow-up, this technique provides acceptable mid-term outcomes with durable structural stability.

## Graphical abstract

**Figure:**
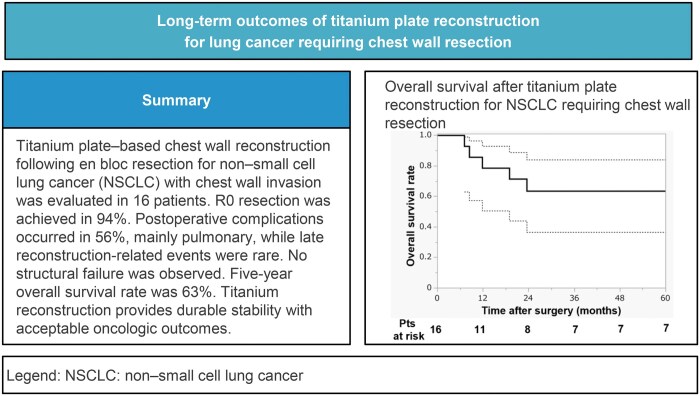

## INTRODUCTION

Lung cancer remains the leading cause of cancer-related mortality worldwide. For patients with stage I to resectable stage III non-small cell lung cancer (NSCLC), multimodal treatment including surgical resection is recommended,^1–3^ and en bloc resection is performed for selected cases with chest wall invasion. Chest wall resection requires not only oncologic clearance but also functional reconstruction to maintain stability and respiratory mechanics. According to a nationwide survey in Japan, such procedures account for approximately 0.5% of lung cancer surgeries, underscoring their rarity.^4^ However, rigid reconstruction remains technically demanding, and optimal strategies—particularly for posterior defects adjacent to the scapula—have not been fully established.

When large portions of the chest wall are resected, prosthetic materials are required to restore rigidity and respiratory mechanics. Although indications for reconstruction are relatively well defined, optimal reconstructive materials remain unclear, and titanium-based devices have been increasingly reported.^5^ Nevertheless, data focusing exclusively on primary lung cancer, particularly with respect to posterior chest wall reconstruction, mid- or long-term structural safety, and late complications, remain scarce. Posterior chest wall defects adjacent to the scapula pose a unique functional challenge, as inadequate reconstruction may lead to scapular-related mechanical complications.^6^ However, the role of rigid reconstruction in this context has not been systematically evaluated.

At our institution, titanium plate-based chest wall reconstruction has been used for primary lung cancer requiring extensive resection. This study aimed to evaluate short- and mid-term outcomes, focusing on posterior reconstruction adjacent to the scapula and structural safety.

## METHODS

### Study design and patients

This single-centre retrospective study included 16 patients who underwent combined chest wall resection and titanium-plate reconstruction for primary lung cancer between 2013 and 2019. Clinical data, operative findings, postoperative course, and oncologic outcomes were collected and analysed. Tumour staging was assessed using the 7th edition of the UICC TNM classification.^7^ The study protocol was approved by the institutional review board (number 2025-68); the need for individual consent was waived owing to the retrospective design.

### Indications for rigid reconstruction and surgical technique

In our hospital, large chest wall defects—defined as resection of ≥3 ribs or a wide 2-rib segment in the lower ribs associated with potential chest wall instability—were generally considered indications for rigid chest wall reconstruction using titanium plates, based on preoperative discussion and intraoperative surgical judgement. In addition, rigid reconstruction was also considered for defects located in areas where structural stability is critical, including the anterolateral and posterior upper chest wall, particularly when there was a risk of postoperative flail chest or scapular impingement. In contrast, for smaller defects or when sufficient chest wall stability was anticipated, non-rigid reconstruction using an ePTFE sheet alone or no reconstruction was considered. No strict institutional protocol was applied, and decisions were made by the attending surgeon based on tumour extent and intraoperative findings. A surgical margin of at least 1 cm was generally required; however, when the margin was anticipated to be closer, intraoperative frozen section analysis was performed to confirm negative margins. Following chest wall resection, 1-3 perforated titanium plates were used to restore chest wall rigidity. We used 2.6 mm thickness straight or angled titanium perforated plates of the Lorenz Mandibular Plating System (Zimmer-Biomet, catalogue number 24-4512, 24-4518, 24-4524, 54-4542, 24-4543) for chest wall reconstruction. Fixation was achieved with titanium wires and sutures instead of screws. Plate configuration (straight or angled) and orientation (cranio-caudal or oblique) were selected according to defect geometry and available fixation, with intraoperative contouring as needed. Angled plates were used when vertebral-side fixation was limited, with limbs oriented to provide lateral and medial support. The plates were fixed to the ribs using titanium wires or thick nonabsorbable sutures. The titanium plate construct was then covered and quilted with a 2-mm ePTFE sheet, most often DualMesh (W. L. Gore & Associates), although standard ePTFE sheets were also available. Finally, skin and soft tissues were closed over drains according to standard practice. In the present study, the topology of the chest wall was determined by dividing the rib arch on CT into 3 equal portions, designated as anterior, lateral, and posterior, according to the centre of the tumour area most adjacent to the chest wall.

### Indications for preoperative chemoradiotherapy

In superior sulcus tumours (SST), resection following preoperative chemoradiotherapy (CRT) has been established as the standard approach based on previous reports. At our institution, we have also actively employed induction CRT for chest wall–invasive lung cancers other than SST. However, patients with poor general condition or those with cavitary tumours carrying a high risk of infection were not considered suitable candidates for CRT. Final treatment strategies were determined at a multidisciplinary cancer board. Decisions regarding postoperative adjuvant chemotherapy were also individualized according to patient condition and discussed at the cancer board.

### Follow-up and outcome definitions

Patients were followed postoperatively with chest or chest–abdominal CT scans every 6 months, along with measurement of tumour markers at 6-month intervals. Outcomes measured in the present study were postoperative complications (≤30 days) and mid-term device-related events. Oncologic endpoints including R0 resection rate, relapse-free survival (RFS), and overall survival (OS) were also analysed. Relapse-free survival was defined as the time from surgery to recurrence or death, whichever occurred first; patients without events were censored at last follow-up. One patient had microscopically positive margins (R1 resection); however, this case was included in the recurrence-free survival (RFS) analysis. Overall survival was defined as the time from surgery to death from any cause, with surviving patients censored at last follow-up. Late complications were defined as events occurring >90 days postoperatively and attributable to the reconstruction or chest wall. Postoperative complications were graded according to the Clavien–Dindo classification.^8^

## STATISTICAL ANALYSIS

Continuous variables are summarized as means or medians with ranges; categorical variables as counts and percentages. Survival was estimated using the Kaplan–Meier method. Statistical analyses were performed using JMP version 18 (SAS Institute Inc.).

## RESULTS

### Patient and tumour characteristics, surgical and pathological factors

Patient characteristics, surgical, and pathological factors are summarized in **Table 1**. All patients underwent major pulmonary resection with mediastinal nodal dissection. Combined pulmonary and chest wall resection achieved an R0 resection rate of 94%. In 1 patient, microscopic residual disease was identified in the connective tissue surrounding the subclavian artery, corresponding to an R1 resection (Case 15 in **Table 2**).

**Table 1.** Patient Characteristics, Surgical, and Pathological Factors

Factors	*n* = 16
Age, years—mean (range)	62.4 (42-77)
Sex	
Male	14 (87%)
Female	2 (13%)
Smoking status	
Never smoker	0 (0%)
Current smoker	7 (44%)
Ex-smoker	9 (56%)
Brinkman index—median (range)	780 (630-2280)
Tumour size, mm—mean (range)	55 (31-75)
Clinical stage (UICC 7th)	
IIA	1 (6%)
IIB	10 (62%)
IIIA	3 (19%)
IIIB	2 (13%)
Preoperative treatment	8 (50%)
Surgical approach	
Posterolateral thoracotomy	13 (81%)
U-shaped incision	2 (13%)
Axillary incision	1 (6%)
Pulmonary resection	
Right upper lobectomy	10 (62%)
Left upper lobectomy	3 (19%)
Right lower lobectomy	2 (13%)
Left pneumonectomy	1 (6%)
Nodal dissection	
ND2a-1	8 (50%)
ND2a-2	8 (50%)
Number of resected ribs	
2 ribs	3 (19%)
3 ribs	9 (56%)
4 ribs	4 (25%)
Transverse process resection, *n* (%)	2 (12%)
Operative time, min—mean ± SD	351 ± 91
Operative time, min—range	207-532
Blood loss, g—mean ± SD	748 ± 456
Blood loss, g—range	150-1670
Transfusion, *n* (%)	8 (50%)
Pathologic stage (UICC 7th)	
pCR	1 (6%)
IIB	8 (50%)
IIIA	5 (31%)
IIIB	1 (6%)
IV	1 (6%)
Histologic type	
Adenocarcinoma	7 (44%)
Squamous cell carcinoma	5 (31%)
Pleomorphic carcinoma	2 (13%)
Adenosquamous carcinoma	1 (6%)
Large cell carcinoma	1 (6%)
Adjuvant chemotherapy, *n* (%)	6 (38%)

**Table 2.** Factors Associated With Chest Wall Reconstruction and Postoperative Complications

Pt number	Age	Sex	Preoperative treatment	Type of resection	Topology of the resected chest wall	Chest wall resection extent	Method of placement of titanium plate	Reconstruction adjacent to scapula	Postoperative 30-day complications
1	56	M	CRT	RUL	Posterior	2nd, 3rd, 4th, 5th ribs	Oblique 1 straight plate	Yes	None
2	55	F	No	RUL	Lateral	2nd, 3rd, 4th ribs	Transverse 1 straight plate	Yes	None
3	57	M	No	LP	Posterior	6th, 7th, 8th ribs	Cranio-caudal 1 straight plate	Yes	Empyema
4	76	M	No	RUL	Lateral	3rd, 4th, 5th ribs	Cranio-caudal 3 straight plates	Yes	Pneumonia
5	42	M	CRT	RUL	Posterior	2nd, 3rd, 4th ribs	angled 1 plate	Yes	Empyema
6	62	M	No	RUL	Posterior	3rd, 4th, 5th ribs	Cranio-caudal 2 straight plates	Yes	None
7	66	M	No	RUL	Anterior	2nd, 3rd, 4th ribs	Cranio-caudal 2 straight plates	No	None
8	62	M	No	LUL	Lateral	3rd, 4th, 5th ribs	Oblique 1 straight plate	No	None
9	62	M	CRT	RUL	Posterior	2nd, 3rd, 4th, 5th ribs	Cranio-caudal 2 straight plates	Yes	Pneumonia
10	73	F	No	LUL	Posterior	2nd, 3rd, 4th, 5th ribs	Cranio-caudal 1 straight plate	Yes	None
11	67	M	CRT	RUL	Posterior	1st, 2nd, 3rd, 4th ribs	angled 1 plate	Yes	Pleural effusion
12	57	M	CRT	LUL	Posterior	2nd, 3rd, 4th ribs	angled 1 plate	Yes	Prolonged air leak
13	57	M	CRT	RUL	Lateral	1st, 2nd, 3rd ribs	angled 1 plate	Yes	Empyema
14	68	M	CRT	RLL	Posterior	9th, 10th ribs	Cranio-caudal 2 straight plates	No	Prolonged air leak
15	77	M	CT	RUL	Anterior	2nd, 3rd ribs	Oblique 1 straight plate	Yes	Atelectasis
16	62	M	No	RLL	Posterior	8th, 9th ribs	Cranio-caudal 2 plates	No	None

Abbreviations: CRT = chemoradiotherapy, F = female, M = male, LP = left pneumonectomy, LUL = left upper lobectomy, RLL = right lower lobectomy, RUL = right upper lobectomy.

### Chest wall reconstruction and postoperative complications

**Figure 1.**
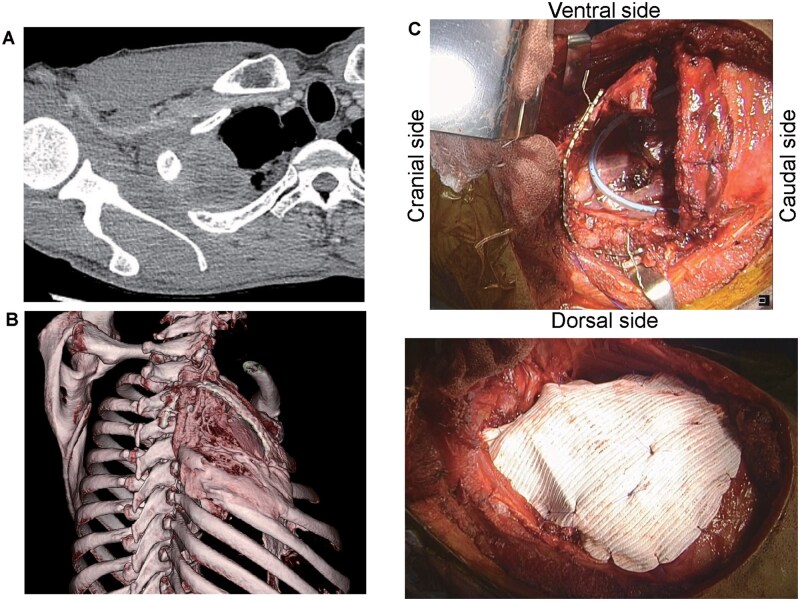
(A) Pre-Treatment Computed Tomography (CT) Revealed a 5-cm Mass in the Right Upper Lobe with Invasion of the First through Third Ribs. (B) Computed tomography (CT) with 3D reconstruction performed after 106 months, demonstrating the reconstructed chest wall (right lateral view). (C) Upper panel: Representative intraoperative view of the reconstruction technique in Case 13. The lateral portions of the first, second, and third ribs were resected en bloc with the right upper lobe. Because the vertebral-side stumps of the second and third ribs were short, placement of a straight plate along the rib axis was difficult. Therefore, an angled plate was used: the short limb was fixed to the vertebral stumps of the second and third ribs with titanium wires, while the long limb was aligned with and fixed to the second rib. Lower panel: A DualMesh was overlaid, with the smooth surface facing the thoracic cavity.

Factors associated with chest wall reconstruction and postoperative complications are summarized in **Table 3**. A representative case of titanium plate-based reconstruction is shown in **Figure 1B and C** (Case 13). Chest wall defects were predominantly located in the posterior region, frequently involving the scapular motion zone. In 12 patients (75%), reconstruction was performed adjacent to the scapula.

**Table 3.** Published Reports of Titanium-Based Chest Wall Reconstruction Since 2010

Author	Year	Study period	Total number of patients	Disease (Lung cancer/other tumour/no tumour resection)	Device/construct	Postoperative complication rate	Mid- or long- term structural failure	Late infection	Follow-up period
Iarussi^17^	2010	Not available	13	3/5/5	Titanium plates (Synthes)+ ePTFE DualMesh	3/13 (23%)	Not available	Not available	Not available
Berthet^10^	2011	2006-2010	19	11/8/0	Titanium devices (STRATOS/VEPTR) + ePTFE DualMesh	3/19 (16%)	Not available	Not available	Mid-term
Berthet^11^	2012	2006-2011	31	13/18/0	Titanium devices (STRATOS/VEPTR) + ePTFE DualMesh	5/31 (16%)	4/31(7%)	0/31 (0%)	Mid-term
Berthet^12^	2015	2009-2013	54	12/17/25	Titanium bars/plates (STRATOS, MatrixRIB)	5/54 (9%)	24/54 (44%)	Not available	Mean 20 months
Yang^18^	2015	2009-2014	27	4/23/0	Titanium mesh (Timesh)	4/27 (15%)	0/27 (0%)	0/27 (0%)	Mean 30 months
De Palma^13^	2016	2010-2014	27	5/6/16	Synthes/MatrixRIB plates ± mesh	12/27(44%)	3/27 (11%)	0/27 (0%)	Median 20 months
Ong^14^	2018	2014-2015	8	3/2/3	MatrixRIB plates + porcine dermal collagen patch (Permacol)	1/8 (13%)	0/8(0%)	0/8(0%)	up to 12 months
Tamburini^15^	2019	2014-2018	26	8/10/8	Titanium mesh (MDF medica)	5/26(19%)	1/26 (4%)	0/26(0%)	Median 21 months
Maniscalco^16^	2020	2015-2018	6	2/1/3	Titanium mesh (MDF medica)	1/6 (17%)	Not available	Not available	Not available
Clermidy^19^	2022	2012-2018	68	8/60/0	Titanium bars (Thorib)/sternal plates (Trionyx)	39/68(57%)	11/68 (16%)	1/68(1%)	Median 34 months
Wong^24^	2022	2011-2020	11	5/4/2	Titanium plates (eg, MatrixRIB) ± mesh/flaps	8/11 (73%)	1/11 (9%)	0/11 (0%)	Mean 57 months
Yoon^25^	2024	2018-2021	5	0/3/2	3D-printed pure titanium implant	0/5 (0%)	1/5 (20%)	1/5 (20%)	Median 20 months
Current study	2026	2013-2019	16	16/0/0	Perforated titanium plates + ePTFE DualMesh	9/16 (56%)	0/16 (0%)	1/16 (6%)	Median 24 months

Rigid reconstruction was achieved using perforated titanium plates. Plate orientation and number were selected according to defect geometry and residual rib length, most commonly in a cranio-caudal configuration. In cases with limited vertebral-side fixation, angled or obliquely oriented plates were used to achieve stable medial fixation, allowing secure restoration of chest wall rigidity across a range of defect configurations. Postoperative complications within 30 days occurred in 9 patients (56%), most of which were pulmonary in nature, including empyema, pneumonia, and prolonged air leak. One patient required postoperative home oxygen therapy because of severely impaired preoperative pulmonary function (ppoDLco <40%) and was therefore not classified as having a postoperative complication.

### Mid-term outcomes

**Figure 2.**
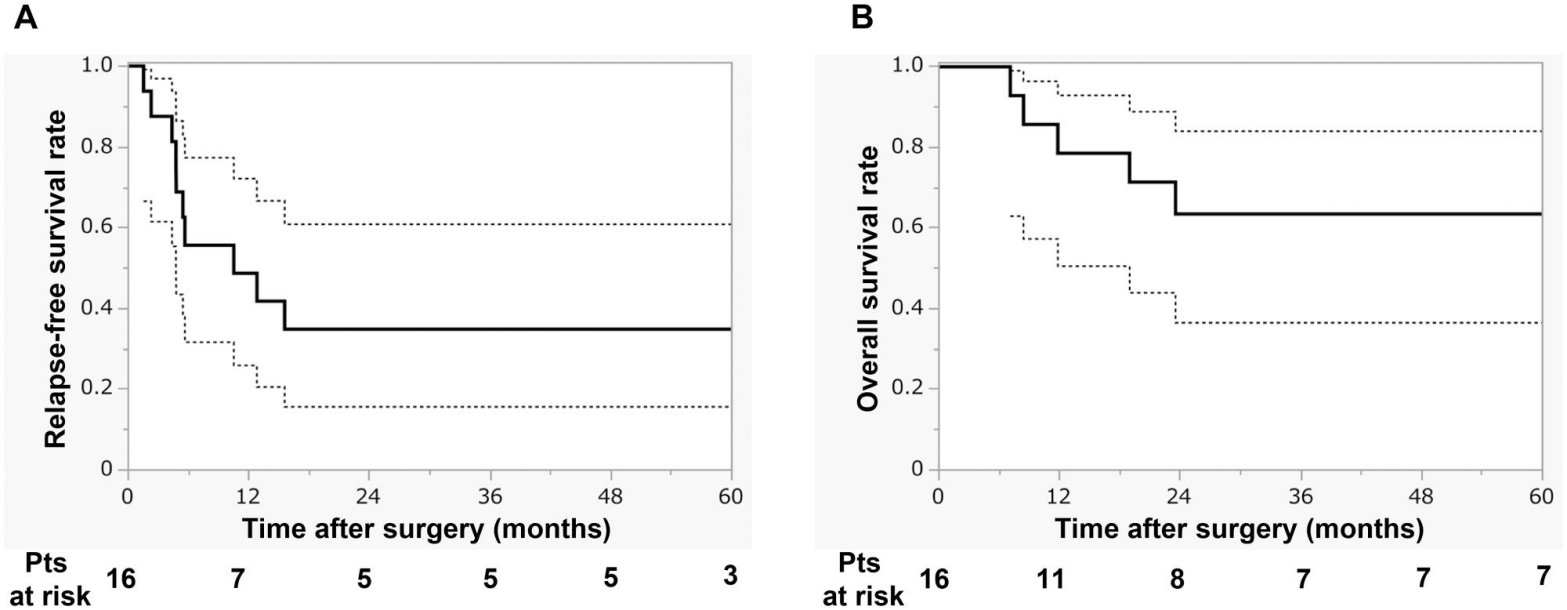
Mid-Term Outcomes. Kaplan–Meier Curves for Relapse-Free Survival (A) and Overall Survival (B). Bold lines indicate Kaplan–Meier estimates and thin lines the 95% CIs.

With a mean and median follow-up of 54 and 24 months (range, 2-147), 10 patients experienced recurrence (local, *n* = 3, including 1 chest wall stump recurrence; distant, *n* = 6; both local and distant, *n* = 1), and 6 patients died. Five-year RFS and OS were 35% (95% CI, 15%-61%) and 63% (95% CI, 37%-84%), respectively (**Figure 2**). Two late complications were recorded (chest wall haematoma at 28 months in Case 1 and empyema at 14 months in Case 9). No structural failure was observed.

## DISCUSSION

This study demonstrated favourable mid-term outcomes of titanium plate-based chest wall reconstruction for primary lung cancer, with en bloc resection of up to 4 ribs achieving a high R0 rate (94%) without structural failure.

Chest wall resection requires complete oncologic resection and postoperative mechanical stability. Various reconstructive strategies—including mesh-based repair, muscle flaps, and rigid fixation—have been reported. Although muscle flaps offer advantages in infection resistance, their limited mechanical strength restricts their applicability in extensive defects. Conventionally, rigid reconstruction is considered unnecessary for posterior upper rib (above 4th rib) defects.^9^ In our practice, however, rigid reconstruction has also been applied to selected upper rib resections based on a broader functional concept. Rigid reconstruction was applied based on a conceptual rationale, including the maintenance of chest wall stability, reduction of paradoxical motion, and potential improvement in postoperative comfort and respiratory mechanics. However, these potential benefits were not objectively evaluated in the present study and therefore remain hypothetical. Accordingly, rigid reconstruction was selectively employed to enhance chest wall stability and safety, although its functional advantages should be interpreted with caution. In cases involving posterior resection at the level of the fifth rib within the scapular excursion zone, rigid reconstruction was additionally intended to prevent scapular impingement. In the present series, reconstruction adjacent to the scapula was performed in 12 patients, including 5 high-risk cases involving posterior fifth-rib resection. No scapular impingement or locking occurred. The upper limit of chest wall resection in this study was 4 ribs, likely reflecting the practical boundary of surgically resectable disease in locally advanced lung cancer.

We summarized reports published since 2010 describing chest wall reconstruction using titanium-based devices in **Table 3**. Previous studies have reported heterogeneous indications and reconstruction strategies, often including non-lung cancer cases.^10–17^ Therefore, direct comparison with the present study, which focuses exclusively on primary lung cancer, is limited. Various titanium-based systems with adjunctive materials have shown acceptable structural outcomes.^5^^,^^10–17^ Postoperative complication rates vary widely in the literature due to heterogeneity in patient populations and surgical extent.^10–19^ The complication rate in our series should be interpreted in the context of extensive combined resections. Importantly, no mid-term structural failure was observed, and late complications were limited, suggesting acceptable durability of the reconstruction.^10–12^^,^^18^^,^^19^

Recurrences in our cohort were clustered within the first 12-15 months after surgery, followed by a plateau in both RFS and OS curves. This pattern may reflect heterogeneity in tumour biology. Patients with early recurrence likely had occult micrometastatic disease at the time of surgery, whereas those without early recurrence may represent a subgroup with more localized disease and more favourable tumour biology. In our cohort, 5 patients remained recurrence-free beyond 60 months, which is consistent with the plateau observed in the Kaplan–Meier curve. Among these patients, 2 had received preoperative CRT, 3 had squamous cell carcinoma, and 2 received postoperative adjuvant therapy. The pathological stages were IIB in 2 patients, IIIA in 2, and IV in 1. When stratified by histology, 3 of 5 patients with squamous cell carcinoma achieved long-term RFS, compared with 2 of 11 patients with non-squamous histology. This may suggest that durable survival can be achieved after complete resection in selected patients with squamous cell carcinoma, even in locally advanced disease. However, given the small sample size, no statistical analysis was feasible, and this observation should be interpreted with caution. A similar pattern observed in OS suggests that early systemic progression is a key determinant of prognosis in this population.

Historically, the 5-year OS rate after resection for chest wall–invading lung cancer has been reported to be approximately 40%-45%, indicating that a substantial proportion of patients experience recurrence despite complete resection.^20^ In a prospective multi-institutional study of induction CRT followed by surgery, approximately one-third of patients developed recurrence,^21^ despite improved survival outcomes in selected patients, and CRT remains the standard treatment for SSTs. In the present study, approximately half of the patients received preoperative therapy, and our oncologic outcomes appear to fall between those reported in these previous studies. With respect to the extent of resection, detailed reporting of the number of resected ribs is limited in the literature. In a representative study by Chapelier et al.,^22^ resection of 3 or more ribs was associated with worse survival, suggesting that patients requiring more extensive chest wall resection may represent a subgroup with more advanced disease and poorer prognosis. In contrast, the majority of patients in our cohort required resection of 3 or more ribs, indicating that our series includes a relatively advanced subgroup in terms of local tumour extent and tumour biology. Importantly, most recurrences in our series were distant rather than local, with only 1 case of chest wall recurrence. This finding suggests that the primary limitation of treatment in locally advanced lung cancer is systemic disease progression rather than inadequate local control or reconstruction strategy. Therefore, the relatively high recurrence rate observed in our cohort is likely attributable to tumour biology and case selection rather than the surgical or reconstructive technique itself. The predominance of male patients in this cohort is consistent with previous reports of NSCLC with chest wall invasion, in which approximately 85%-90% of patients were male.^20^^,^^21^ This may partly reflect the epidemiology of smoking-related lung cancer and differences in tumour growth patterns and anatomical distribution. However, the marked predominance of male patients cannot be fully explained, and further investigation is warranted. In addition, selection bias cannot be excluded, as patients undergoing extensive chest wall resection and reconstruction represent a highly selected subgroup with locally advanced but surgically resectable disease.

With recent advances in perioperative systemic therapy, preoperative immune checkpoint inhibitor (ICI)-based regimens have shown promising efficacy and are increasingly adopted for chest wall–invading lung cancers other than SSTs.^23^ As chest wall resection was permitted in a recent pivotal trial,^23^ an important future challenge will be to establish the feasibility and safety of chest wall resection followed by rigid reconstruction after preoperative ICI-based therapy.

This study has several limitations. This retrospective single-institution study is subject to selection bias and limited generalizability. The small sample size reflects the rarity of the procedure and limits statistical robustness. The absence of a non-rigid reconstruction control group precludes direct comparison with alternative techniques. In addition, reconstruction strategies were determined based on clinical judgement, which may further introduce selection bias. The lack of systematic postoperative functional assessment, including pulmonary function testing and patient-reported outcomes, precludes definitive conclusions regarding the functional necessity of rigid reconstruction, particularly for upper rib defects. Therefore, the potential functional advantages of rigid reconstruction should be considered hypothesis-generating. Furthermore, its superiority over alternative reconstruction methods cannot be established due to the lack of comparative data, and the possibility of overtreatment in selected cases cannot be excluded. Future studies using multicentre cohorts and comparative designs are warranted to validate the generalizability and clinical relevance of these findings. Finally, detailed surgical factors such as the extent of rib resection are inconsistently reported in the literature, making it difficult to directly compare the invasiveness of our cohort with previously published series.

## CONCLUSIONS

Titanium plate-based chest wall reconstruction enables extensive en bloc resection for NSCLC with chest wall invasion while maintaining mid-term structural stability. With appropriate patient selection and careful follow-up, this technique provides acceptable mid-term outcomes with durable structural stability.

## Data Availability

The datasets used and/or analysed during the current study are available from the corresponding author upon reasonable request.
